# Cell membrane array fabrication and assay technology

**DOI:** 10.1186/1472-6750-5-18

**Published:** 2005-06-16

**Authors:** Victoria Yamazaki, Oksana Sirenko, Robert J Schafer, Luat Nguyen, Thomas Gutsmann, Lore Brade, Jay T Groves

**Affiliations:** 1 Synamem Corporation, 863 Mitten Road – Suite 101, Burlingame, CA94010, USA; 2 Division of Medical and Biochemical Microbiology, Research Center Borstel, Center for Medicine and Biosciences, Borstel, Germany; 3 Department of Chemistry, 109 Lewis Hall, University of California – Berkeley, Berkeley, CA 94720, USA

## Abstract

**Background:**

Microarray technology has been used extensively over the past 10 years for assessing gene expression, and has facilitated precise genetic profiling of everything from tumors to small molecule drugs. By contrast, arraying cell membranes in a manner which preserves their ability to mediate biochemical processes has been considerably more difficult.

**Results:**

In this article, we describe a novel technology for generating cell membrane microarrays for performing high throughput biology. Our robotically-arrayed supported membranes are physiologically fluid, a critical property which differentiates this technology from other previous membrane systems and makes it useful for studying cellular processes on an industrialized scale. Membrane array elements consist of a solid substrate, above which resides a fluid supported lipid bilayer containing biologically-active molecules of interest. Incorporation of transmembrane proteins into the arrayed membranes enables the study of ligand/receptor binding, as well as interactions with live intact cells. The fluidity of these molecules in the planar lipid bilayer facilitates dimerization and other higher order interactions necessary for biological signaling events. In order to demonstrate the utility of our fluid membrane array technology to ligand/receptor studies, we investigated the multivalent binding of the cholera toxin B-subunit (CTB) to the membrane ganglioside GM_1_. We have also displayed a number of *bona fide *drug targets, including bacterial endotoxin (also referred to as lipopolysaccharide (LPS)) and membrane proteins important in T cell activation.

**Conclusion:**

We have demonstrated the applicability of our fluid cell membrane array technology to both academic research applications and industrial drug discovery. Our technology facilitates the study of ligand/receptor interactions and cell-cell signaling, providing rich qualitative and quantitative information.

## Background

DNA and protein microarrays have enabled the measurement of gene expression and biomarkers on an unprecedented scale, and have greatly affected fields ranging from oncology to medical diagnostics. However, assaying biological events occurring on cell membranes, including signal transduction and intercellular communication, has proven to be considerably more difficult to industrialize in microarray format. Basic methods for reconstituting membrane proteins into phospholipid vesicles were developed quite a number of years ago [1], facilitating fundamental discoveries in such diverse cellular processes as metabolism, solute transport, protein trafficking, and signal transduction. In subsequent years, it was demonstrated that planar phospholipid bilayers could be generated simply by fusing these vesicles to various solid substrates (e.g. glass [2] and quartz [3]), generating "supported membranes" which have lateral fluidity. The characteristic fluidity, uniformity of lipid/protein orientation, and planarity make these supported membranes ideal for studying most membrane components, including receptors, transporters, channels, and adhesive factors. Alternatively, planar lipid bilayers can be deposited on surfaces derivatized with various polymeric films [4-6]. Regardless of how the planar membrane interacts with the solid support, display of these lipid bilayers represents a powerful way of developing technologies ranging from biosensors [4,7] to platforms for the study of ion channel electrophysiology [8]. Given the increasing complexity of current basic research experiments and industrial drug discovery schemes, the ability to pattern arrays of lipid bilayers has become almost essential for performing multiplexed, high information-content assays. To address this need, there have been a number of methods developed over the past few years to pattern arrays of lipid bilayers on solid supports for purposes of studying the biology of membranes and their component lipids and proteins [9-12]. While these unique approaches all generate patterns of lipid bilayers on solid supports, they vary greatly in a number of important ways, including the manner of lipid bilayer display (e.g. supported above or directly associated with a polymer linked to the solid support) and the means of generating the membrane pattern (e.g. lithographic modification of the solid support to allow selective deposition of membranes [9,10,12,13] or modification of the planar bilayer following deposition [11]).

**Figure 1:**
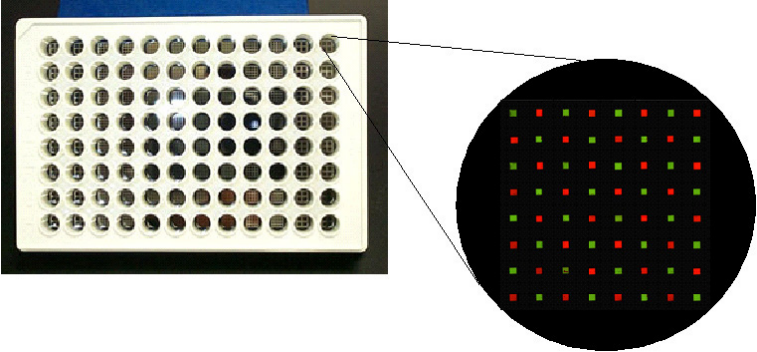
**Schematic diagram of cell membrane arrays for drug discovery. **The 64 250-μm × 250-μm corrals are separated by 750 μm of chrome which prevents contamination of supported membranes from one corral to another. Each corral contains a supported lipid bilayer membrane which displays different molecules or different concentrations of the same molecule. The array elements are subsequently exposed to ligands or live cells, allowing for observation of differences in binding or cell biological events among the corrals. Each array resides in a single well of a 96-well plate, facilitating multiplexed assays.

We have recently developed a technology for industrializing physiologically fluid supported membrane microarrays relying upon the discovery that fused silica, normally a compatible substrate for supported lipid bilayers, can readily be made membrane-incompatible through various lithographic procedures [9,13]. This development led to the realization that fluid cell membranes could be isolated in independently-addressable corrals on a silicon chip through lithographic patterning. In essence, microarrays consisting of spatially-indexed corrals of supported lipid bilayers can be generated at very high density (Figure 1) [14]. Membrane corrals are regions of a solid substrate, in this case fused silica, which are compatible with lipid bilayer deposition and are isolated from each other by membrane-barriers composed of chrome. In this manner, cell membranes are supported on a silicon chip with an aqueous interface between the membrane and solid support, as well as a bulk aqueous phase above the membrane [9,13,14]. The isolated membranes in each corral of the array are physiologically fluid as confirmed by fluorescence recovery after photobleaching (FRAP). In this technique, excitation light shadows a small region of the corral, photobleaching the fluorescent molecules in that area. Following this excitation step, the photobleached region of the corral is examined for a recovery of fluorescence which is dependent upon the ability of fluorescent lipids in the surrounding membrane to diffuse into the photobleached area. The extent of membrane fluidity determines the rate at which these fluorescent molecules diffuse into the photobleached area and diminish the shadow formed by the excitation step. Another technique used to measure the fluidity of supported lipid bilayers is the lateral migration of charged lipids in the presence of an electric field [9,13,14]. Given their aqueous surroundings, both upper and lower bilayers of the membrane exhibit a physiological level of fluidity. The fluidity of our supported lipid bilayer arrays permits interactions among membrane components (e.g. oligomerization of cell surface receptors) on a physiological time scale, a phenomenon thought to underlie numerous signal transduction pathways. This report demonstrates that the individually-corralled lipid bilayers can faithfully mimic the structural and functional characteristics of *in vivo *cell membranes, making them valuable for the study of biological processes ranging from simple ligand/receptor interactions to complex cell-cell signaling.

As mentioned previously, fluidity is a critical property of biological membranes *in vivo*. According to biophysical models of membrane fluidity, there is a logarithmic dependence of the diffusion coefficient of a given molecule (e.g. a membrane protein) on its radius, a phenomenon enabling large proteins embedded in the membrane to diffuse nearly as rapidly as lipid molecules [15]. The fluidity dynamics are completely different for membranes interacting directly with solid substrates, where friction greatly attenuates the diffusion of larger molecules (e.g. proteins) in comparison to lipids [15]. The characteristic fluidity of supported membranes depends entirely upon the manner in which the lipid bilayer is prepared and the type of solid substrate supporting the membrane. In order to retain such a high level of fluidity, these membranes cannot be exposed to an air-water interface. Integrated membrane technologies, in which lipid bilayers are deposited on a self-assembled chemical monolayer coating the solid substrate instead of directly on the substrate itself, have sought to eliminate this drawback. However, the increased convenience of coating the substrate with a chemical monolayer is offset by the observation that integrated membranes have considerably reduced fluidity (approximately 40% less) in comparison to supported lipid bilayers deposited directly on bare glass [6]. Consequently, the supported membranes described in this study display a level of fluidity more characteristic of the *in vivo *environment, a property likely to be critical for numerous cell biological events.

Fluid membrane protein arrays are thus a powerful technology for displaying lipid bilayers which facilitates the study of cell surface processes. Planar synthetic membranes and their molecular components are readily displayed in a fluid and functional state characteristic of the *in vivo *environment. While the basic principles of fluid bilayer arrays were invented in academia [9,13], we have industrialized the technology to a level suitable for high throughput drug screening. By combining lipid biochemistry with semiconductor microfabrication and robotic handling, membranes are arrayed at high density in discrete corrals on a silicon chip. In order to complement the membrane array, we have also developed methods for efficiently expressing, purifying, and displaying the vast majority of membrane proteins in the genome. Taken together, the membrane array and membrane protein display technologies enable the study of biological processes occurring on cell membranes. In this article, we report the validation of our membrane array technology for studies pertaining to ligand/receptor binding and live cell-based assays. We initially characterized our supported lipid bilayers by displaying glycolipid molecules found in mammalian (ganglioside GM_1_) and bacterial (LPS) cells. We have chosen to study the interaction between CTB and the membrane ganglioside GM_1_. Cholera toxin, naturally secreted by the bacterium *Vibrio cholerae*, is thought to exist as a hexamer consisting of two subunits in an AB_5 _configuration. The pentameric CTB subunit is thought to bind up to five GM_1_gangliosides in a cooperative fashion [16,17]. This multivalent interaction likely requires membrane fluidity for proper stoichiometric assembly of the ligand/receptor complex. Additionally, we demonstrate the successful display of various other membrane components, including LPS from Gram-negative bacteria and the mammalian proteins ICAM-1 and I-E^k^, molecules which are important drug targets for treating diseases ranging from septic shock in the case of the former to autoimmunity in the case of the latter. Gram-negative bacteria display LPS in the outer membrane of their cell wall. Also referred to as endotoxin, LPS consists of two distinct regions: a hydrophilic polysaccharide moiety and the highly conserved hydrophobic toxin lipid A [18,19]. As LPS is present only in bacterial membranes, it has been a target of continual interest to drug discovery programs aimed at developing more efficacious antibiotics. Our studies examining T cell adhesion and activation on membrane protein arrays displaying immunological proteins demonstrates the utility of our platform for biological assays. In these T cell experiments, we have displayed proteins present on antigen presenting cells which are critical to generating an immunological response: the adhesion protein ICAM-1 and the class II dimeric antigen-presentation molecule I-E^k^. The ability to study complex multivalent ligand/receptor interactions and the engagement of live cells with our supported lipid bilayers could aid not only industrial drug screening against membrane targets, but also basic research studies in academia.

## Results

### Arraying high quality supported membranes

**Figure 2:**
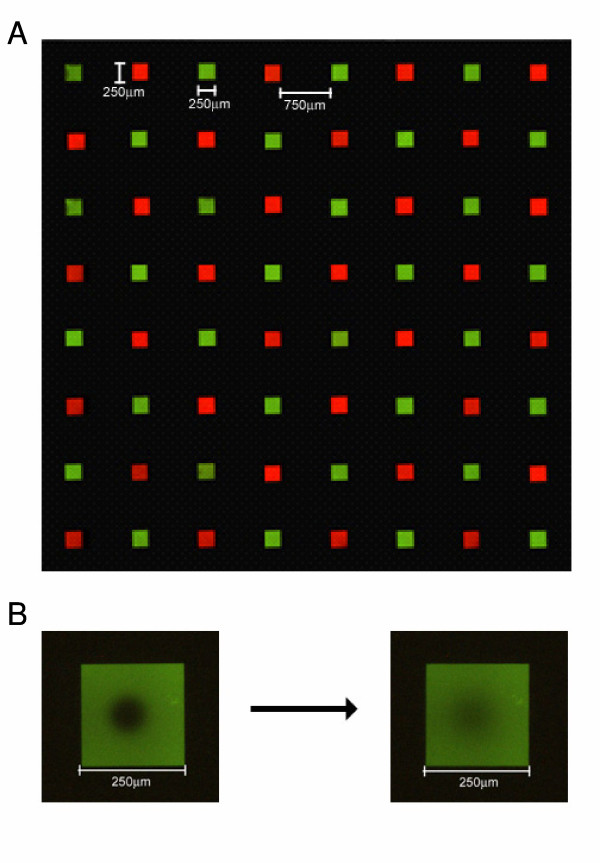
**Independently addressable cell membrane arrays. **(A) An 8 × 8 64-corral supported membrane array containing different molecules (1 mol % NBD-PG or 1 mol % Texas Red DHPE in egg PC) in an alternating pattern was arrayed robotically in a manner which prevents mixing of contents from corral to corral. (B) Photobleaching a region of membrane containing 1 mol % NBD-PG in egg PC is followed by a recovery.

**Figure 3:**
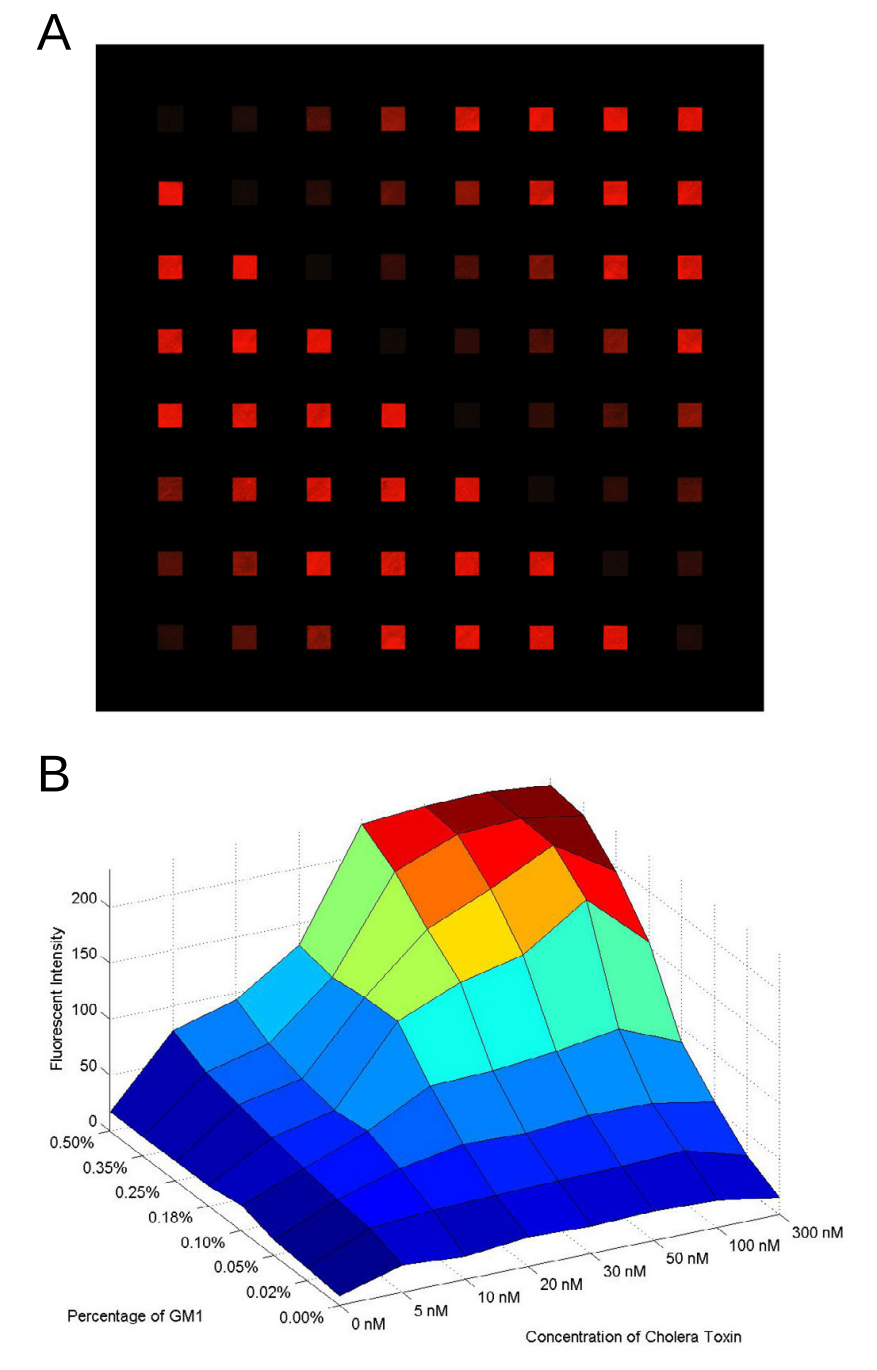
**A single-pass high information-content binding curve of the CTB/GM_1 _interaction. **(A) 8 different concentrations of ganglioside GM_1 _(0.00, 0.02, 0.05, 0.10, 0.18, 0.25, 0.35 and 0.50 mol %) are microarrayed in the pattern of an arrow. The chips were then exposed to a fluorescently-labeled CTB, revealing the variation in GM_1 _concentration. (B) This experiment was performed on 8 different supported membrane chips. Using the microarrayer, the eight 8 × 8 chips were prepared with the same distribution of ganglioside GM_1_. They were subsequently exposed to different concentrations of fluorescently-labeled CTB. The arrays were imaged microscopically and fluorescence was quantitated using Adobe Photoshop.

We sought to characterize the quality and precision of our fluid membrane arraying capabilities for industrial production. The CTB/GM_1 _interaction afforded a system to study our supported lipid bilayers, as its pentavalent structure is likely to require a high degree of membrane fluidity for efficient complex formation. As depicted in Figure 2A, membranes containing unique lipid compositions (red- or green-fluorescent dopant lipids in this case) were arrayed at very high density in a manner which prevents mixing of contents from corral to corral. Moreover, the arrayed membranes were of high quality in terms of their physiological level of fluidity, as determined by FRAP measurements (Figure 2B). In order to demonstrate the precision of arraying membranes of varying compositions into specific corrals of an array, we used a pre-written program (8 × 8 arrow pattern with 8 samples) to deposit 8 different concentrations of ganglioside GM_1 _on a chip in a manner so as to generate an arrow-shaped pattern (Figure 3A). GM_1 _was detected using fluorescence microscopy following an incubation with fluorescently-labeled CTB (red), a method we have previously used to investigate this ligand/receptor pair [14].

### High information-content binding of CTB to GM_1 _arrayed on supported membranes

Given the CTB/GM_1 _binding illustrated in Figure 3A, we next wished to quantify this ligand/receptor interaction. Figure 3B depicts a binding experiment performed using eight 8 × 8 (64 corral) supported membrane arrays displaying varying concentrations of GM_1_. We used 8 different concentrations of GM_1 _(0.00, 0.02, 0.05, 0.10, 0.18, 0.25, 0.35 and 0.50 mol percent) and 8 different concentrations of CTB (0, 5, 10, 20, 30, 50, 100, and 300 nM). Hence, we performed a ligand binding experiment using 8 replicates for each concentration of CTB applied so as to obtain highly quantitative and information-rich binding data with very little reagent material required. The fluid membranes were arrayed using a pre-written program consisting of an 8 × 8 standard loop with 8 samples. As in the case of the membranes in Figure 2, fluidity was confirmed using the FRAP technique. Figure 3B is a contour plot of the quantitation of the CTB/GM_1 _data, indicating the expected specific and saturable binding of the ligand to its receptor.

### Incorporation of diverse membrane components into supported fluid lipid bilayers and live cell assays

**Figure 4:**
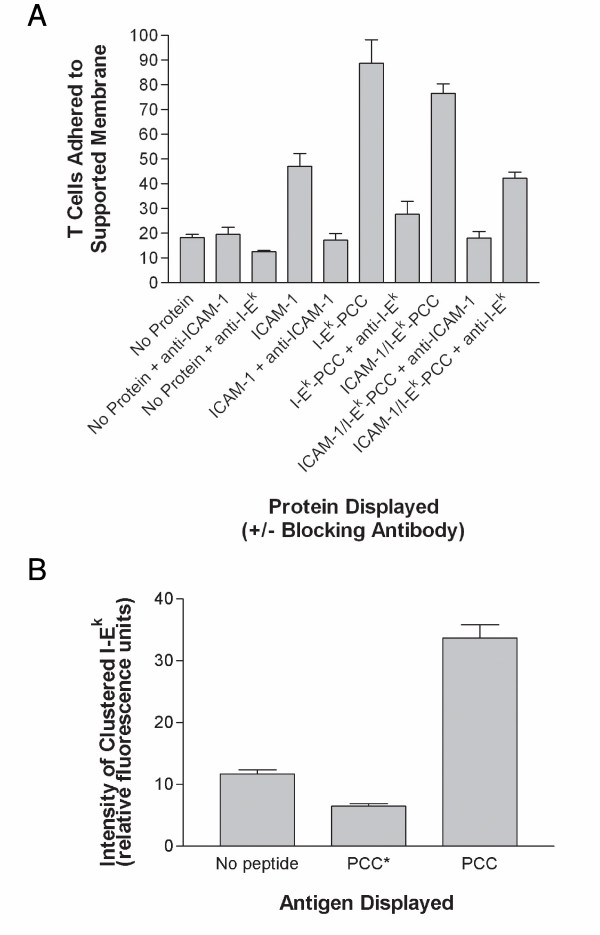
**Live cell assays for academic research and industrial drug discovery. **(A) Control membranes (devoid of protein) or membranes reconstituted with either ICAM-1 or I-E^k^-complexed to PCC peptide alone or together were incubated with murine T cells specific for PCC in the presence or absence of specific antibodies to either protein. Adhered cells were counted under the microscope and averaged from different corrals. (B) Murine T cells specific for PCC were incubated with supported lipid bilayers displaying fluorescently-labeled ICAM-1 and I-E^k ^– complexed to wild-type PCC, a mutated PCC*, or no peptide, and were subsequently assayed for the intensity of clustered I-E^k ^at the center of synapses. Values represent means ± SEM from at least 3 independent determinations.

In order to further validate our system for industrial drug discovery and basic research applications, we have purified and displayed important molecules mediating microbial pathogenesis (LPS) and various immunological disorders (ICAM-1 and I-E^k^). Lipid A, the principal endotoxic moiety of LPS, was displayed in lipid bilayers and detected specifically by the anti-lipid A antibody (A6), yielding an approximately 4-fold increase in immunoreactivity compared with membranes devoid of the endotoxin (data not shown). We next sought to extend our technology for assaying membrane biology important to the immune system. The ectodomains of ICAM-1 (an adhesion molecule) and I-E^k ^(an antigen-presentation molecule) were over-expressed in mammalian cells, purified, and displayed in supported lipid bilayers through glycosylphosphatidylinositol (GPI) tethering (data not shown). Given that these two proteins are particularly important in mediating the adhesion and activation of T cells on antigen-presenting cells, we examined these proteins for their ability to capture a transgenic murine T cell line [20] specific for the pigeon cytochrome c (PCC) antigen. Figure 4A illustrates that surfaces displaying either ICAM-1 or I-E^k^-complexed to PCC as GPI-linked proteins mediate specific adhesion of T cells when displayed either alone or in combination, while membranes devoid of these proteins confer almost no adhesion. Moreover, in order to mimic a potential therapeutic intervention, we show that monoclonal antibodies specific for either ICAM-1 or I-E^k ^nearly abolish T cell adhesion, while having little effect on the low level of non-specific adhesion to membranes devoid of protein (Figure 4A). In addition to measuring adhesion, the physiological level of fluidity of our supported membranes makes them ideal for assaying the full T cell activation process. Following adhesion of T cells to antigen-presenting cells, there is a dramatic relocalization of signaling molecules at the cell-cell interface known as the "immunological synapse," which is thought to be the most proximal marker of antigen-dependent T cell activation [21]. Upon initial encounter of the PCC-specific murine T cells with a membrane displaying fluorescently-labeled ICAM-1 and I-E^k ^complexed to PCC peptide, T cells adhere and form an immature "synapse" characterized by ICAM-1 localized centrally and I-E^k ^at the periphery (data not shown). During the next 30 minutes of T cell engagement, however, this "synaptic" pattern changes dynamically, as the peptide-loaded I-E^k ^migrates centrally and ICAM-1 adopts a more peripheral localization (data not shown). In addition to measuring "immunological synapse" formation qualitatively as has been previously demonstrated [21], we can also obtain quantitative information about this marker of T cell activation by measuring the clustering of cell surface molecules. As presented in Figure 4B, we have quantified the intensity of clustered I-E^k ^to measure the strength of "synapse" formation and, hence, T cell activation. As a control for quantitative measurement, we have compared the strength of "synapse" formation in the presence of I-E^k ^complexed to a wild-type PCC peptide to that complexed to either no peptide or a mutated PCC* peptide which cannot elicit T cell expansion. Figure 4B demonstrates not only the capability of obtaining robust quantitative data for T cell activation using our assay system, but also the high degree of antigen-specificity recorded in the experiment.

## Discussion

We report here the industrialization of membrane protein microarrays suitable for basic research and drug discovery applications in numerous aspects of membrane biology, from multivalent ligand/receptor interactions to live cell assays. Through robotic handling approaches, it is possible to construct high density arrays of supported lipid bilayers containing unlimited unique combinations of membrane components. The experiments illustrated in Figure 2 and Figure 3 underscore the physiological integrity of our membranes as determined by their *in vivo*-like fluidity, as well as the ability to array these membranes at high density without causing mixing of contents among corrals. The high quality of the arrayed membranes is extremely reproducible between corrals and among different chips. Further, the precision of the arraying equipment facilitates deposition of unique membrane compositions in specific corrals of a membrane array. The physiological fluidity of each discretely-arrayed bilayer is truly what enables the study of membrane processes, which often require the dynamic lateral motion of proteins and lipids.

In order to validate our system for studying membrane biology, we first examined the interaction between CTB and GM_1_, a multivalent binding event likely to require fluidity of the bilayer for proper complex formation. As illustrated in Figure 3, a vast amount of high content data can be obtained rapidly (within minutes) for the CTB/GM_1 _system, or nearly any other ligand/receptor system, given the ease with which concentrations of ligand and receptor can be varied and analyzed. This advantage would be extremely beneficial for investigating the pharmacodynamics of essentially any ligand against membrane components. Indeed, the experiment depicted in Figure 3 was performed rapidly and only required 8 wells of a 96-well microtitre plate. Given the physiological level of fluidity in our supported membrane arrays, it is possible to study ligand/receptor interactions in a manner beyond capturing mere binding data. Actual signaling can be examined using our technology, given that the fluid and dynamic motion of membrane components is preserved. We chose the CTB/GM_1 _interaction precisely for its multivalency, a property which may require fluidity of the surrounding lipid membrane environment for proper complex formation. Indeed, pentavalent CTB is thought to recruit five GM_1_molecules in a process that demonstrates positive cooperativity among the components [16,17]. Hence, the characteristic fluidity of our lipid bilayers is what permits the study of CTB/GM_1 _and likely numerous other membrane receptors on an almost *in vivo*-like level. While the CTB/GM_1 _system is instructive for studying multivalent ligand/receptor interactions in the context of fluid membranes, we wanted to validate our technology for studying various other types of membrane components. Our ability to display bacterial LPS could facilitate academic studies in the area of host/pathogen interaction, as well as lead to the identification of novel therapeutics to treat life-threatening septic shock. While the polymyxin family of amphiphilic antibiotics target LPS, there is a continuing push to develop more efficacious anti-endotoxin molecules to treat sepsis [22]. For example, candidate antibody or small molecule therapeutics for septic shock could be identified from studying the interaction of immune system cells with membrane surfaces displaying LPS. In order to validate our technology for live cell-based assays, we have examined the activation of intact T cells on membrane arrays displaying molecules critical for adhesion and antigen-presentation. As in the case of the CTB/GM_1 _interaction, we chose a cell-cell association (in this case T cells and antigen-presenting cells) which would highlight the power of our fluid lipid bilayer technology to faithfully mimic *in vivo *membrane environments. The adhesion of T cells to the supported membranes (Figure 4A) models the first mechanistically-distinct stage of T cell activation, which happens to be an important point for therapeutic intervention in a number of immunological disorders. Indeed, Raptiva, the recently-approved antibody therapeutic for psoriasis, directly targets the ICAM-1/LFA-1-mediated T cell adhesion process [23]. In addition to cell adhesion, our ability to measure full T cell activation in both qualitative and quantitative formats underscores our ability to capture actual signal transduction events occurring on our fluid lipid bilayers, which enable the dynamic reorganization of surface receptors so crucial for this process. While T cell adhesion and partial activation has been demonstrated on arrays consisting solely of MHC deposited on a glass surface without membranes or other molecules present, it is possible that this technology fails to capture important nuances of the activation pathway which may be critical in identifying novel membrane components capable of modulating these processes [24]. In addition, drugs targeting the T cell activation process may have subtle effects which can be distinguished only through qualitative observation of the dynamic "immunological synapse." Studying T cell activation in array format could be quite useful in identifying the roles of various T cell or antigen-presenting cell membrane proteins in this process. Specifically, these proteins could be arrayed in the presence of antigen-presentation machinery in order to assess their precise roles in the distinct stages of T cell activation, including T cell adhesion, T cell stopping, and synapse formation. In essence, activation of live T cells on our supported membranes greatly facilitates high information-content immunological assays.

## Conclusion

Our membrane protein microarray system extends the capabilities of DNA and protein arrays. While the latter have successfully industrialized the analysis of gene and protein expression, fluid cell membrane arrays represent the industrialization of membrane biology. Given that the vast majority of pharmaceuticals, including most of the recent protein- and antibody-based therapeutics, target membrane components, this system has the capability to advance the drug discovery process. Moreover, the technology is also useful for academic research applications, which will undoubtedly form the basis of many future therapeutic opportunities. The combination of physiological *in vivo*-like qualities of our membranes with robotic arraying has created a powerful system for studying membrane biology in a high throughput fashion.

## Methods

### Reagents

Egg phosphatidylcholine (egg-PC) and 1-Palmitoyl-2- [12-[(7-nitro-2-1,3-benzoxadiazol-4-yl)amino]dodecanoyl]-*sn*-Glycero-3- [Phospho-rac-(1-glycerol)] (ammonium salt) (16:0-12:0 NBD-PG) were obtained from Avanti Polar Lipids. N-Texas Red sulfonyl-1,2-dihexadecanoyl-sn-glycero-3-phosphoethanolamine (triethylammonium salt) (Texas Red DHPE) and CTB (recombinant) Alexa Fluor^® ^594 conjugate (labeled CTB) were obtained from Molecular Probes (presently Invitrogen). Unlabeled CTB was purchased from Sigma Chemical Co.

### Fabrication of supported membrane arrays

Micropatterned substrates were fabricated on glass wafers, micropatterned with chrome barriers following a "piranha clean" (3:1 mixture of sulfuric acid and 30% solution of hydrogen peroxide). Array surfaces were cleaned by soaking for 20 minutes in freshly prepared piranha solution. Subsequently, the surfaces were rinsed under deionized water. The small unilamellar vesicles (SUVs) were prepared by standard extrusion methods. Briefly, lipids were mixed in the desired concentrations in chloroform, followed by evaporation of the chloroform in a round bottom flask using a rotary evaporator (Buchi, Rotovap R200). The lipids were resuspended in distilled water and stored overnight. Following hydration overnight at 4°C, they were passed 21 times through an Avanti extruder containing a membrane with 100-nm pores. The extruded vesicles were stored at 4°C until use within several weeks.

Planar supported bilayers were formed by fusion of SUVs onto the piranha-cleaned surfaces. Prior to supported membrane deposition, spreading solutions were prepared by mixing the SUV suspensions in equal ratios with phosphate-buffered saline (PBS). The fabrication of lipid microarrays was carried out using a microarrayer (Cartesian technologies model MycroSys™ SQ series) equipped with software (AxSys™ software) for programmable aspiration and dispensing. Due to the very small drop size (nanoliters), the microarrayer was enclosed in a humidified chamber (~98% humidity) at room temperature to avoid evaporation. In order to prevent contamination from carryover between different lipid solutions, an automatic wash cycle was incorporated into every arraying program that consisted of consecutive washes in deionized water and dry cleaning before each sample aspiration. Following the arraying step, the arrayed elements were submerged in deionized water. They were then washed 3 times with PBS, followed by incubation with CTB in PBS on a rocker (set at 2.75) for one hour at room temperature. Subsequently, they were washed 6 times with PBS on the rocker (set at 2.75) with 5-minute washes for the last three.

### Reconstitution of LPS into SUVs

The pathogenic moiety of LPS (lipid A) was isolated from R595 LPS (LPS from deep rough mutant of *Salmonella enterica *Minnesota strain R595) by sodium acetate buffer treatment (0.1 M, pH 4.4, 100°C for 1 hour) and was dissolved in a 65:25:4 ratio mixture of chloroform:methanol:deionized water. Multilamellar vesicle formation was performed using standard methods. Briefly, the solvent was evaporated from the lipid mixture using a rotary evaporator (Buchi Rotary Evaporators, C Assembly). The remaining lipid cake was hydrated overnight at 4°C to form multilamellar vesicles. Multilamellar vesicles were sonicated in order to increase the incorporation of LPS into unilamellar vesicles, followed by extrusion to isolate the SUV population. The monoclonal IgG2b anti-lipid A antibody (A6) was prepared as previously described [25]. The goat anti-mouse IgG (H&L) antibody conjugated to Cy2 was purchased from Jackson ImmunoResearch Laboratories. Standard immunofluorescence protocols as recommended by Jackson Immuno were followed.

### Generation and display of ICAM-1 and I-E^k^

cDNAs encoding the extracellular domains (excluding signal sequences) of human ICAM-1 and murine I-E^k ^(alpha and beta chains) were amplified from ESTs (Invitrogen) and subcloned into pcDNA3.1 (Invitrogen) modified to encode a fused in-frame signal sequence and His6 sequence in tandem at the N-terminus and a GPI-specifying sequence at the C-terminus (pYBS101) as *Hind*III-*Xba*I fragments with enterkinase-encoding cleavage sites (DDDDK) at their N-termini to generate proteins having a C-terminal GPI anchor. CHO cells were transfected with expression plasmids using calcium phosphate transfection (Promega) or SuperFect (Qiagen) following manufacturers' recommendations. 48 hours after transfection, cells were split at 1:5, 1:10 and 1:20 into growth media containing 500 μg/ml of G418 (Invitrogen) for generation of stable lines expressing human ICAM-1 or murine I-E^k^. Polyclonal stable cell lines were maintained and passaged in G418-containing media for 4 weeks. Expression of these proteins was confirmed by FACS analysis using PE-conjugated antibodies (Becton Dickinson). Confluent CHO cells from 6–8 10-cm dishes were washed 2X with cold PBS, and were scraped into 600 μL (per dish) of lysis buffer containing 50 mM Tris-Cl pH 8.0, 150 mM NaCl, and 1% Triton X-100, and a protease inhibitor cocktail (Sigma). Harvested cells were solubilized on ice for 30 minutes, followed by microcentrifugation for 15 min at the highest speed. Ni-NTA agarose (Qiagen) was washed 1X with lysis buffer and added to clarified extracts (50 μL of beads per 600 uL of cell extract). Cell extracts were incubated with beads for 2 hours with rotation at 4°C. Beads were washed 4X with buffer containing 50 mM Tris-Cl pH 8.0, 150 mM NaCl, 10 mM imidazole (Sigma), and 1% Octyl-Glucopyranoside (Calbiochem) plus a protease inhibitor cocktail (Calbiochem). Bound proteins were eluted with 600 μL of buffer containing 50 mM Tris-Cl pH 8.0, 150 mM NaCl, 200 mM imidazole, and 1% Octyl-Glucopyranoside plus a protease inhibitor cocktail for 1 hour at 4°C with rotation. Aliquots of protein extracts were added to an egg phosphatidylcholine (PC) lipid mix (1% NBD-PG, 99% egg PC) and dialyzed overnight at room temperature against PBS, with 3X buffer changes. While egg PC was used to generate membranes in this case, these proteins could easily be reconstituted in vesicles with various lipid compositions. To form the supported lipid membranes, 20 μL of undiluted (or diluted 1:2) protein-lipid mix was deposited onto silicon chips or cover slips for 3–5 min at room temperature, followed by 5 washes with buffer. Incorporation of protein into the supported membrane was confirmed by immunofluorescence using antibodies to the respective protein. Membranes were stained with 30 μL of PE-conjugated antibodies (BD Pharmingen) in 5% fetal calf serum (FCS) for 30 min, and then washed extensively with PBS and assessed by fluorescence microscopy. Protein densities are approximately 10,000–20,000 molecules/μm^2^, as estimated from a proteolipid concentration of 0.6 μg/μL (50% pure) in a 20-μL drop on a chip surface of 7 mm diameter. It was assumed that only 1% of the drop contents is retained on the chip surface for membrane display.

### T cell isolation, adhesion, and immunological synapse formation

Murine T cells specific for PCC were derived from spleens harvested from the T cell receptor transgenic mouse strains B10.Cg-Tg(TcrAND)53Hed/J and B6;SJL-Tg(TcrAND)53Hed/JCell (The Jackson Laboratory). T cells were isolated from splenocytes by magnetic bead selection (Dynal) according to the manufacturer's instructions. T cell adhesion assays to supported membranes displaying either ICAM-1 or I-E^k ^as GPI-linked proteins were performed as previously described with several modifications [26]. Briefly, membranes were blocked with 10% fetal bovine serum (FBS) in PBS for 1 hour at room temperature, followed by incubation for 10 minutes with RPMI 1640 media (Invitrogen) containing 10% FBS at 37°C. Approximately 1 × 10^5 ^T cells were added to the well for 15 minutes. Following incubation with cells, corrals were washed for 10–15 minutes in the presence of PBS with 5% serum. Where appropriate, blocking antibodies were used at an approximately 1:100 dilution. The approximate number of cells per membrane corral was determined by counting under a brightfield microscope (usually 150–200 cells/corral). Supported lipid bilayers were subsequently inverted into a dish containing 10% FBS in PBS for 15–20 minutes to wash unbound cells. The final cell number was determined by counting under microscope directly or after imaging. In experiments where monoclonal antibodies against human ICAM-1 or murine I-E^k ^(Santa Cruz) were used to block T cell adhesion, supported membranes were pre-incubated with antibody followed by 3 washes with PBS prior to incubation with cells. "Immunological synapse" formation experiments were performed as described previously [21]. Imaging was performed on a Nikon Eclipse 300 vertical microscope, and analysis and quantitation were performed using Adobe Photoshop.

### Imaging of membrane arrays

Imaging of the membrane microarrays was performed on a Nikon Eclipse 300 vertical microscope using a 4 × objective. Fluorescence images were taken with the same objective in fluorescence mode. The supported membranes were fluorescently labeled with 1 mol % of NBD, Texas Red-tagged lipids or labeled CTB. Images were recorded with a digital color camera driven by CoolSnap software. In cases where it was not possible to image the entire chip in one field (e.g. a 64 corral chip), the image was reconstructed in piecemeal fashion. We used Adobe Photoshop software to measure the intensity of each corral and Microsoft Excel to analyze the data.

## Authors' contributions

VY performed the endotoxin experiments and oversaw the completion of all other experiments described in this manuscript. LN contributed the arrays depicted in Figure 2 and Figure 3A, and RS performed the CTB/GM_1 _experiment shown in the quantitation in Figure 3B. OS performed the immunological experiments depicted in Figure 4. TG provided endotoxin and LB provided the lipid A monoclonal antibodies. JG provided advice throughout the course of this work.
